# PAIN EXPERIENCES IN OLDER ADULTS WITH HEART FAILURE IN THE HOME HEALTHCARE SETTING AFTER HOSPITAL DISCHARGE

**DOI:** 10.1093/geroni/igz038.976

**Published:** 2019-11-08

**Authors:** Youjeong Kang, Cary Reid

**Affiliations:** 1 University of Utah, Salt Lake City, Utah, United States; 2 Weill Cornell Medicine, New York, New York, United States

## Abstract

Pain symptoms are underreported in older adults (i.e., those ages ≥65), because many consider it a part of aging. Pain is highly prevalent in patients with heart failure (HF). HF is increasing in incidence with the aging population. Pain is also an important risk factor for rehospitalization among home healthcare patients with HF. However, little is known about the pain experiences of older adults with HF after hospital discharge. Accordingly, this study sought to describe pain and other symptoms among older adults with HF in the home healthcare setting using a mixed-methods approach. We conducted semi-structured interviews. We used the Edmonton Symptom Assessment Scale and Brief Pain Instrument on older adults (N=17) with HF in the home healthcare setting within 10 days of hospital discharge. The mean age was 76±9.25 years, nearly 50% were female, and most (90%) were White. The majority of participants managed pain with medications. Loss of appetite had the highest mean severity score (3.88±2.50) of symptoms measured. The mean score for pain at its worst was 4.94±3.07. Pain interfering with sleep had the highest mean score (4.24±3.36). Using content analysis we identified 4 distinct themes regarding the pain experience: (1) differences in the pain experiences before and after being diagnosed of HF, (2) symptoms accompanied with pain, (3) health care providers’ pain assessment, and (4) knowledge about pain management. These findings will contribute to the development and evaluation of a pain assessment and management protocol targeting older adults with HF in the home healthcare setting.

